# Supplementary material for: Age of initiation of hookah use among young adults: Findings from the Population Assessment of Tobacco and Health (PATH) study, 2013–2017
Source: PLoS One. 2021 Oct 12;16(10):e0258422. doi: 10.1371/journal.pone.0258422 (PMC8509879; doi:10.1371/journal.pone.0258422)
Supplement: S1 Table — (DOCX) [file pone.0258422.s001.docx]

**S1 Table. Frequency distribution of ever use of other tobacco products prior to past 30-day hookah use and fairly regular hookah use.**

| **Variable** | | | **N=6,631; N=22,669,597** | |
| --- | --- | --- | --- | --- |
|  |  |  | **Unweighted n (N)** | **Weighted % (SE)** |
| **Other tobacco product use prior to past 30-day hookah use** | Cigarette | No | 4,285 (16,060,851) | 70.8% (0.95) |
|  |  | Yes | 2,346 (6,608,746) | 29.2% (0.95) |
|  | E-cigarette | No | 4,622 (17,431,140) | 76.9% (0.80) |
|  |  | Yes | 2,009 (5,238,457) | 23.1% (0.80) |
|  | Smokeless Tobacco | No | 6,030 (21,084,456) | 93.0% (0.40) |
|  |  | Yes | 601 (1,585,141) | 7.0% (0.40) |
|  | Traditional Cigars | No | 5,774 (20,171,048) | 89.0% (0.55) |
|  |  | Yes | 857 (2,498,548) | 11.0% (0.55) |
|  | Filtered Cigars | No | 5,938 (20,876,620) | 92.1% (0.40) |
|  |  | Yes | 693 (1,792,976) | 7.9% (0.40) |
|  | Cigarillo | No | 5,023 (18,317,155) | 80.8% (0.65) |
|  |  | Yes | 1,608 (4,352,442) | 19.2% (0.65) |
| **Other tobacco product use prior to fairly regular hookah use** | Cigarette | No | 4,254 (15,931,237) | 70.3% (0.97) |
|  |  | Yes | 2,377 (6,738,359) | 29.7% (0.97) |
|  | E-cigarette | No | 4,545 (17,154,730) | 75.7% (0.79) |
|  |  | Yes | 2,086 (5,514,867) | 24.3% (0.79) |
|  | Smokeless Tobacco | No | 6,019 (21,053,646) | 92.9% (0.40) |
|  |  | Yes | 612 (1,615,951) | 7.1% (0.40) |
|  | Traditional Cigars | No | 5,728 (20,040,672) | 88.4% (0.55) |
|  |  | Yes | 903 (2,628,924) | 11.6% (0.55) |
|  | Filtered Cigars | No | 5,904 (20,787,399) | 91.7% (0.42) |
|  |  | Yes | 727 (1,882,198) | 8.3% (0.42) |
|  | Cigarillo | No | 4,993 (18,221,287) | 80.4% (0.66) |
|  |  | Yes | 1,638 (4,448,310) | 19.6% (0.66) |

¥ PATH restricted file received disclosure to publish: March 29, 2021. United States Department of Health and Human Services. National Institutes of Health. National Institute on Drug Abuse, and United States Department of Health and Human Services. Food and Drug Administration. Center for Tobacco Products. Population Assessment of Tobacco and Health (PATH) Study [United States] Restricted-Use Files. ICPSR36231-v13.AnnArbor, MI: Inter-university Consortium for Political and Social Research [distributor], November 5, 2019. <https://doi.org/10.3886/ICPSR36231.v23>.

SE: Standard error.
