# Supplementary material for: Age of initiation of hookah use among young adults: Findings from the Population Assessment of Tobacco and Health (PATH) study, 2013–2017
Source: PLoS One. 2021 Oct 12;16(10):e0258422. doi: 10.1371/journal.pone.0258422 (PMC8509879; doi:10.1371/journal.pone.0258422)
Supplement: Attachment — Submitted filename: Reviewers_Comments_08_30_2021.docx [file pone.0258422.s002.docx]

Answer to the Academic Editor:

1. Please ensure that your manuscript meets PLOS ONE's style requirements, including those for file naming. The PLOS ONE style templates can be found at <https://journals.plos.org/plosone/s/file?id=wjVg/PLOSOne_formatting_sample_main_body.pdf> and <https://journals.plos.org/plosone/s/file?id=ba62/PLOSOne_formatting_sample_title_authors_affiliations.pdf>.

**Response:** Thank you for this comment. We reviewed all the requirements again and we did the following changes:

1. Symbol * is added in page 1 to indicate corresponding author.
2. (Maximum: 300 words) next to Abstract title was removed.
3. Space is created between level 3 headings and the texts in the Outcomes section.
4. Figure titles are bolded and the square brackets are removed from the titles.
5. The font size for the section title References is changed to 18pt and only the first letter is capitalized.
6. Supporting table title is bolded and is moved to supporting information section created at the end of the manuscript.

2. If materials, methods, and protocols are well established, authors may cite articles where those protocols are described in detail, but your submission should include sufficient information to be understood independent of these references (<https://journals.plos.org/plosone/s/submission-guidelines#loc-materials-and-methods>). As such, please ensure that your details on the PATH study are sufficiently well-described external to any previous literature on this research.

**Response:** In lines 81-82 of the Methods section with track changes, we included the following statement.

“The PATH study is an ongoing nationally representative longitudinal cohort study of U.S. youth (ages 12-17) and adults (ages 18+) conducted annually since wave 1 2013-2014, collecting information from the participants about their demographics, tobacco use behaviors, substance use behaviors, beliefs and attitudes toward tobacco use, and tobacco-related health outcomes”

3. We note that the grant information you provided in the ‘Funding Information’ and ‘Financial Disclosure’ sections do not match. When you resubmit, please ensure that you provide the correct grant numbers for the awards you received for your study in the ‘Funding Information’ section.

**Response:** The grant number under NIH and under FDA are the same as both institutions granted the funding. When this is included in the online system it does not allow us to include both as one institution or a full sentence. The only option was to include each funding entity individually. For this reason, is the discrepancy which we do not know how to solved to agree in your online system. We would like to keep the full sentence:

**Funding acknowledgements**: Research reported in this publication was supported by grant number [R01-CA2342051] from the National Cancer Institute (NCI) and the FDA Center for Tobacco Products (CTP) to Dr. Adriana Pérez. The content is solely the responsibility of the authors and does not necessarily represent the official views of the National Institutes of Health (NIH) or the Food and Drug Administration (FDA).

4. In your Data Availability statement, you have not specified where the minimal data set underlying the results described in your manuscript can be found. PLOS defines a study's minimal data set as the underlying data used to reach the conclusions drawn in the manuscript and any additional data required to replicate the reported study findings in their entirety. All PLOS journals require that the minimal data set be made fully available. For more information about our data policy, please see <http://journals.plos.org/plosone/s/data-availability>.

**Response:** The PATH study does not allow to share restricted data. For this reason, we made modifications in our data availability statement as follows:

**Data Availability Statement**

All the data from waves 1-4 are available from the Population Assessment of Tobacco and Health (PATH) Study [United States] Restricted-Use Files. Inter-university Consortium for Political and Social Research [distributor], 2020-06-24. <https://doi.org/10.3886/ICPSR36231.v27>. Researchers can apply for access to the restricted-use datasets from the Inter-university Consortium for Political and Social Research (ICPSR) at the University of Michigan. To access data in the Virtual Data Enclave (VDE), a Restricted Data Use Agreement (RDUA) must be established between the University of Michigan and the researcher's institution. Data are provided via ICPSR's VDE. For further information, please reference the VDE Guide to learn about the application process, about using the VDE, and how to request disclosure review of VDE output located here: <https://www.icpsr.umich.edu/web/pages/NAHDAP/vde/index.html>

Obtaining results using the restricted-use datasets requires a disclosure process with protocols set by ICPSR. When a researcher logs on to the VDE, a virtual machine is launched on the researcher's own desktop but operates from a server at ICPSR. The virtual machine is isolated from the researcher's physical desktop computer - users cannot download or upload files or parts of files from or to the VDE; print VDE contents to a printer; or email, copy, or otherwise move files in or out of the VDE computing environment, either accidentally or intentionally. Results are only disclosed by ICPSR after programs have been checked for accuracy and results have been replicated.

**Answers to reviewers’ comments**

Reviewer #1:

The authors submit a retrospective study using restricted use files from PATH to analyze the association between age and intensity of hookah use initiation among different demographic groups as a step toward understanding where to target future education interventions. This is a worthwhile study, the manuscript is well written, and the analysis seems robust. However, I have concerns about the novelty of the results given how much has been published on the topic to date. Please note that our study is on the age of hookah initiation behaviors, which have never been reported before. While there are many studies on hookah incidence or prevalence, what is missing from these previous studies is the age at which young adults initiate hookah behaviors.

| Data for youth (<18 years old) exists in PATH so I am curious why age 18 was chosen at the cut off. My assumption is that children initiate hookah use prior to age 18 and earlier targeted intervention may be even more beneficial. Why was this age group not included? | In a previous publication, we published that young adults reported higher proportions of initiation than youth after one year of follow up:  https://www.ncbi.nlm.nih.gov/pmc/articles/PMC6362999/  In addition, we have already published a paper (<https://pubmed.ncbi.nlm.nih.gov/33545623/>) on the age of initiation of hookah use outcomes in youth where participants were 12-17 years old when they did not use hookah and the age of initiation after 4 years of followed up was estimated. In this manuscript, we wanted to determine the age of initiation of hookah use among young adults who did not try hookah, when they entered the PATH study (were18-24 years) but who were followed-up up to 4 years. We believe these are two different subpopulations, as young adults were legally able to access hookah at the time of the data collection. |
| --- | --- |
| For the other tobacco product variable –your data suggest a differential influence of certain other tobacco products (Table 6) on your outcome. I can’t seem to find how and where this variable was used and if it accounts for that. Accordingly, was this variable treated as ordinal or categorical in whichever models it was included? | Without track changes, lines 121-126 describe how the other tobacco product use variables were created. Lines 172-179 describe how the Cox models for each of the age of initiation of the three hookah use outcomes were created as covariates. These variables accounted for prior use of each tobacco product separately instead of the “number of other tobacco products ever used” variables in Table 2.  In addition, please note that each variable for “ever tobacco product use prior to hookah initiation” are binary variables. For example, the variable for “ever cigarette use prior to hookah initiation” contains the responses “yes” and “no”, which is reported in Table 6. Again, we have added the description of these variables in line 121. |
| Given the somewhat vague and subjective wording regarding “fairly regular” use, perhaps some mention of this as a limitation to understanding true intensity of use would be beneficial in the discussion | We agree with the reviewer that “fairly regular” hookah use is a subjective measure, and we have included this as limitation in the strengths and limitations section (Without track changes, lines 429-431). |
| I’m confused about how this study “prospectively estimates” initiation of hookah use. I realize your results can be predictive or prognostic but the word prospective seems incorrect. | Without track changes, lines 64-67 introduce the goal of the study, which is to estimate the age of hookah initiation outcomes among never users of hookah. Given that we start with never users (waves 1-3 at their first wave of participation in the PATH adult study), and examine the age of hookah initiation in follow-up waves (2-4), we are calculating the upper and lower age bound for the interval-censored age outcomes (ever, past 30-day, and fairly regular hookah use) prospectively in waves 2-4. The probability of initiation at a particular age for each outcome is estimated using weighted interval-censoring survival analyses. For this reason, we believe we are using the terminology correctly. Without track changes, lines 85-90 provide more details on our analytic sample, which consists of never hookah users in order to estimate the age of hookah initiation outcomes prospectively. |
| The discussion would be improved with some added insight into how and why your results differ from prior studies and some additional framing of the importance of your results. | It should be noted that our study examines the AGE of initiation and there are no prior studies on the age of hookah initiation in young adults, so we can only compare our results with previous studies of incidence and prevalence. With track changes, lines 358-361 describe how our study is different from previous studies of hookah use. We have included some additional information that speculates on differences between our results and the results of previous research (Lines 370-373, Lines 375-377, Lines 390-394). Lines 395-402 and 420-425 provide some additional framing of the importance of the study results. |
| Similarly, why do you believe there differences between this study’s results and prior studies that have also used PATH data? | Previous studies of PATH data have examined the prevalence or incidence of hookah use in a single wave of data, while our study examines the **age** of hookah initiation using multiple follow-up waves. This is described in the discussion of the current manuscript. |

We appreciate the feedback of the manuscripts which help us to improve the manuscript for readers.
